# Supplementary material for: Silver–Gold Alloy Nanoparticles (AgAu NPs): Photochemical Synthesis of Novel Biocompatible, Bimetallic Alloy Nanoparticles and Study of Their In Vitro Peroxidase Nanozyme Activity
Source: Nanomaterials (Basel). 2023 Sep 1;13(17):2471. doi: 10.3390/nano13172471 (PMC10490118; doi:10.3390/nano13172471)
Supplement: Supplementary file 1 [file nanomaterials-13-02471-s001.zip › nanomaterials-2380843-supplementary.pdf]

Supplementary material

Silver–Gold Alloy Nanoparticles (AgAu NPs): Photochemical Synthesis of Novel Biocompatible, Bimetallic Alloy Nanoparticles and Study of Their In Vitro Peroxidase Nanozyme Activity

Prakash G. Kshirsagar [1,*], Valeria De Matteis [2], Sudipto Pal [3] and Shiv Shankar Sangaru [4,*]

[1] Department of Biochemistry and Molecular Biology, University of Nebraska Medical Center, Omaha, NE 68198, USA
[2] Department of Mathematics and Physics "Ennio De Giorgi", University of Salento, Via Arnesano, 73100 Lecce, Italy
[3] Department of Innovation Engineering, University of Salento, Via Per Monteroni, 73100 Lecce, Italy
[4] Saudi Aramco, Dhahran 31311, Saudi Arabia
* Correspondence: prakash.kshirsagar@unmc.edu (P.G.K.); shiv.sangaru@aramco.com (S.S.S.); Tel.: +1-40-2514-7245 (P.G.K.); +966-513-873-3465 (S.S.S.)

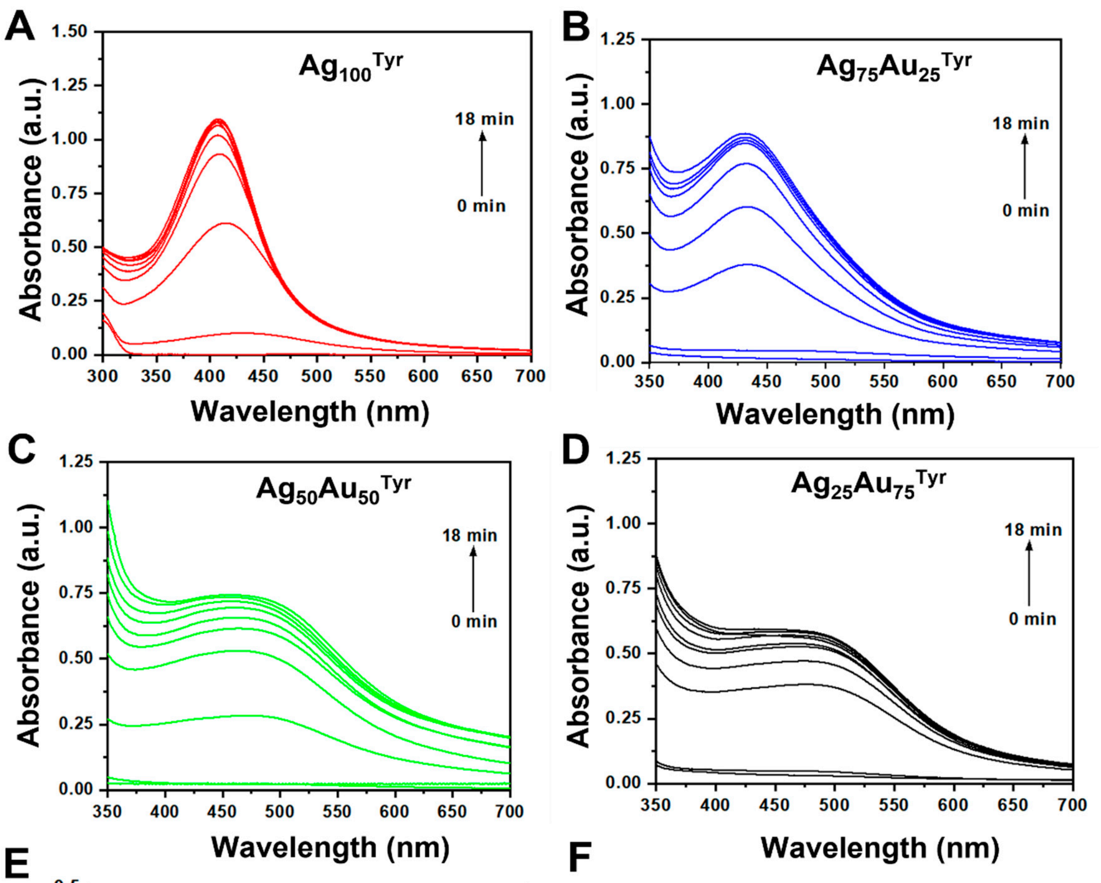

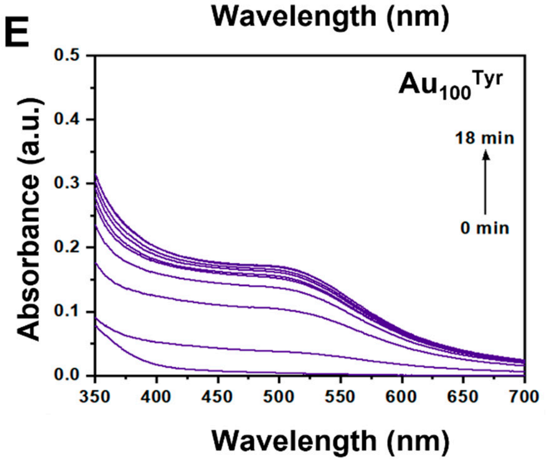

F

Nanoparticle sample	Abs (0 min)	Abs (18 min)	Abs shift	SPR (λ_{max})
Ag_{100}^{Tyr}	0.002	1.094	1.092	408
$Ag_{75}Au_{25}^{Tyr}$	0.020	0.886	0866	430
$Ag_{50}Au_{50}^{Tyr}$	0.025	0.743	0.717	462
$Ag_{25}Au_{75}^{Tyr}$	0.047	0.577	0.530	488
Au_{100}^{Tyr}	0.005	0.168	0.163	510

Figure S1. Time dependent UV visible spectra of Tyrosine-reduced mono/bimetallic NPs. UV visible absorption spectra of mono and bi-metallic NPs during 0-18 min time span. **(A)** Ag_{100}^{Ty}, **(B)** $Au_{75}Au_{25}^{Tyr}$, **(C)** $Au_{50}Au_{50}^{Tyr}$, **(D)** $Ag_{25}Au_{75}^{Tyr}$, **(E)** Au_{100}^{Tyr}**, (F)** Table summarizing results of the time-dependent UV visible study.

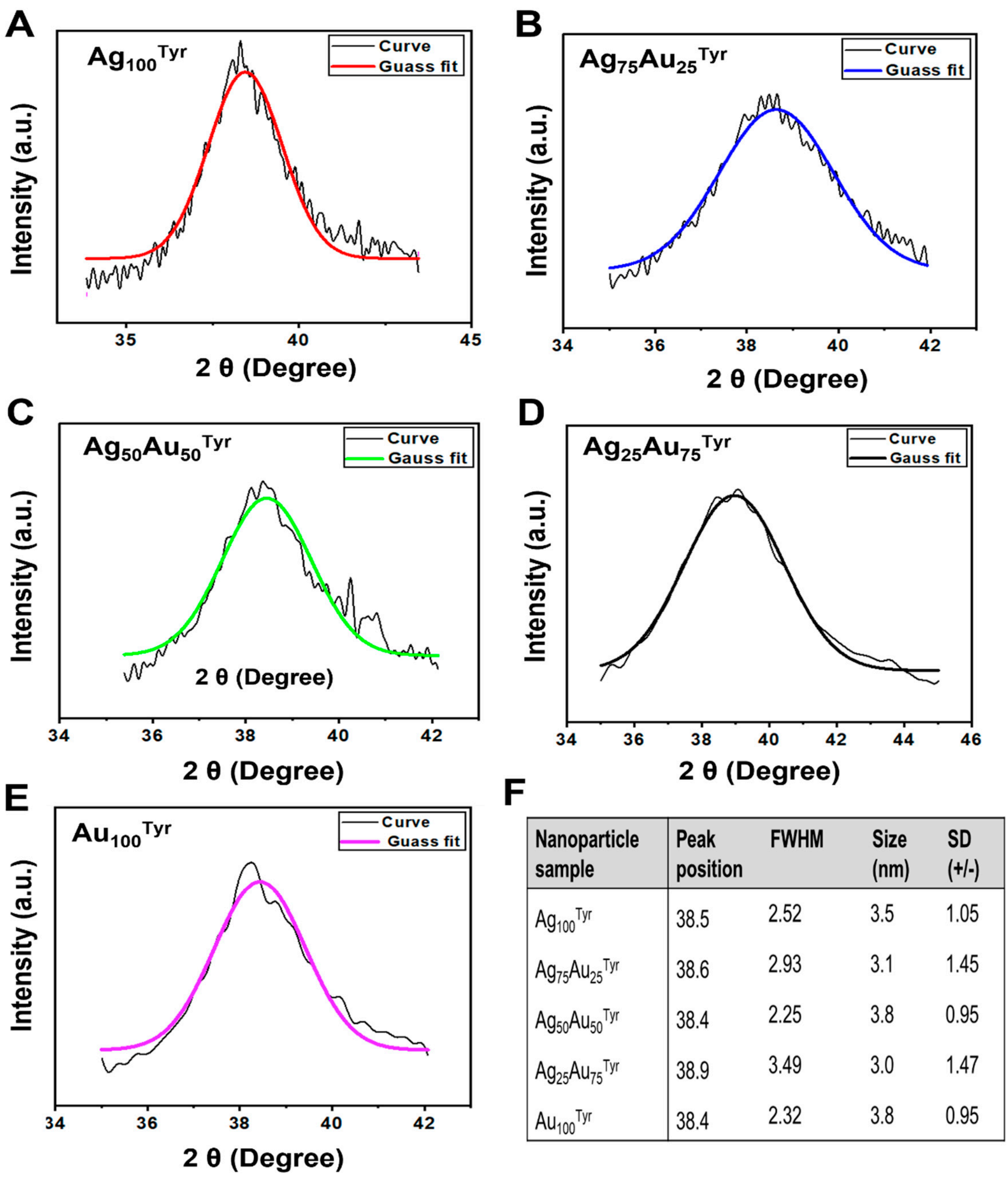

Nanoparticle sample	Peak position	FWHM	Size (nm)	SD (+/-)
Ag_{100}^{Tyr}	38.5	2.52	3.5	1.05
$Ag_{75}Au_{25}^{Tyr}$	38.6	2.93	3.1	1.45
$Ag_{50}Au_{50}^{Tyr}$	38.4	2.25	3.8	0.95
$Ag_{25}Au_{75}^{Tyr}$	38.9	3.49	3.0	1.47
Au_{100}^{Tyr}	38.4	2.32	3.8	0.95

Figure S2. Nanoparticle size determined by the XRD. The nanoparticle diameter (nano-crystal grain size) determined by the Scherrer equation (i.e., fit the XRD peaks and determined the FWHM [2θ] by Gaussian curve fitting). **(A)** Ag_{100}^{Ty}, **(B)** $Au_{75}Au_{25}^{Tyr}$, **(C)** $Au_{50}Au_{50}^{Tyr}$, **(D)** $Ag_{25}Au_{75}^{Tyr}$, **(E)** Au_{100}^{Tyr}, **(F)** Table summarizing results from the XRD study.

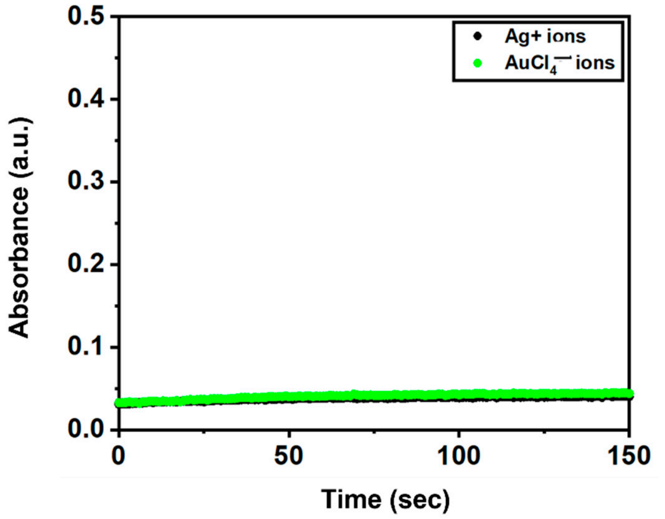

Figure S3. The in vitro peroxidase mimicking activity determined for the Ag^{+}, and $AuCl_4^{-}$ (i.e., control ions).
